# Downregulation of miR-654-3p in Colorectal Cancer Indicates Poor Prognosis and Promotes Cell Proliferation and Invasion by Targeting SRC

**DOI:** 10.3389/fgene.2020.577948

**Published:** 2020-09-30

**Authors:** Haoran Zhang, Zhanlong Shen, Yushi Zhou, Zhen Zhang, Quan Wang, Mengmeng Zhang, Kewei Jiang, Shan Wang, Yingjiang Ye, Bo Wang

**Affiliations:** ^1^Department of Gastroenterological Surgery, Peking University People’s Hospital, Beijing, China; ^2^Laboratory of Surgical Oncology, Peking University People’s Hospital, Beijing, China; ^3^Beijing Key Laboratory of Colorectal Cancer Diagnosis and Treatment Research, Beijing, China

**Keywords:** colorectal cancer, miR-654-3p, SRC, proliferation, invasion

## Abstract

**Background:**

MicroRNAs (miRNAs), such as miR-654-3p, regulate gene expression at the post-transcriptional level affecting malignant tumor behavior. However, the expression levels, function, and mechanism of miR-654-3p in colorectal cancer (CRC) are unknown.

**Methods:**

The expression levels of miR-654-3p and SRC in 103 CRC tissues and matched normal colorectal tissues were detected by a quantitative real-time polymerase chain reaction (qRT-PCR). miR-654-3p was overexpressed by RNA mimics and SRC knockdown by siRNA. Function-based experiments were carried out to detect the proliferation and migration abilities in CRC cell lines. Flow cytometry assay was performed to evaluate the effect of miR-654-3p on cell apoptosis and cycle distribution. Xenograft tumor models in nude mice were utilized to evaluate miR-654-3p functions *in vivo*. Dual-fluorescence reporter assay was used to verify the direct binding between miR-654-3p and SRC.

**Results:**

miR-654-3p was downregulated in CRC tissues as compared to matched normal colorectal tissues. The expression levels of miR-654-3p were closely associated with distant metastasis. In addition, elevated expression of miR-654-3p in CRC patients prolonged the overall survival. Upregulated miR-654-3p significantly suppressed the proliferation and migration capacity of CRC cells by enhancing apoptosis and promoting G0/G1 phase arrest. The direct binding between miR-654-3p and SRC was verified by the dual-luciferase reporter gene. Furthermore, the suppression of proliferation and migration capacity by elevated miR-654-3p level could be reversed by overexpressing SRC.

**Conclusion:**

miR-654-3p acts as a tumor suppressor through regulating SRC. It might also serve as a diagnostic and prognostic indicator and a novel molecular target for CRC therapy.

## Introduction

Colorectal cancer (CRC) is one of the top three malignant neoplasms worldwide, with increasing rates of morbidity and mortality (Siegel et al., 2019). Despite the conspicuous progress made in diagnostic and therapeutic strategies, clinical outcome and prognosis of CRC patients remain poor (Sonbol et al., 2019). Thus, further study on the molecular mechanisms of CRC progression and migration is essential, which might also discover novel therapeutic targets of CRC.

MicroRNAs (miRNAs) are non-coding single-stranded RNA molecules involved in the regulation of post-transcriptional gene expression via binding to 3’-untranslated region (UTR) of mRNAs (Lu and Rothenberg, 2018). Several studies have identified a crucial role of miRNAs in tumor progression and migration (Nair et al., 2012). For instance, the low expression level of miR-490-3p and miR-194-5p significantly promote the proliferation and invasion of CRC cells and lead to poor prognosis for CRC patients (Wang et al., 2018; Wu et al., 2019). Moreover, *miR-654-3p* has been reported as a tumor suppressor gene in hepatocellular carcinoma, gastric cancer, and prostate cancer influencing the progression and migration of the cancer cells (Formosa et al., 2014; Deng et al., 2020; Yang et al., 2020). The role of miR-654-3p in CRC occurrence and development requires further investigation.

SRC family kinases (SFKs) are responsible for aggressive tumor proliferation, migration, and invasion in numerous malignancies (Yeatman, 2004). Also, SRC was elevated and activated in CRC cell lines and CRC tissue, thereby contributing to its malignant behavior (Aligayer et al., 2002; Chen, 2008). Conversely, in recent years, the research on SRC inhibitors has shown unsatisfactory results. For instance, Phase II trials have shown that saracatinib attenuates oxaliplatin uptake in CRC in patients in combination regimens, specifically with 5FU and oxaliplatin (Morrow et al., 2010). Dasatinib plus FOLFOX with or without cetuximab in metastatic CRC failed to inhibit SRC, which indicates meaningless clinical activity in refractory colorectal cancer (Parseghian et al., 2017). The present study is designed to verify whether SRC is directly targeted by miR-654-3p and demonstrate the role of miR-654-3p/SRC pathway in the development of CRC. Simultaneously, the suppression of SRC by miR-654-3p may provide a miRNA-based target for clinical treatment.

## Materials and Methods

### Tissue Specimens

A total of 103 pairs of CRC specimens and matched adjacent normal colorectal tissues were collected from surgeries at Peking University People’s Hospital during January 2013 to December 2015 in subjects that underwent radical resection of CRC. The tissues were kept at −80°C until use. The informed consents were signed by all subjects prior to specimens’ collection. The present study received approval from the Research Ethics Committee of Peking University (Beijing, China). And the following information were obtained: age, sex, tumor size, tumor differentiation, TNM stage, microsatellite stability and survival time.

### Cell Lines

Human colorectal cell lines (NCM460, SW480, RKO, HCT8, HCT116, and lovo) were purchased from National Infrastructure of Cell Line Resource of China. SW480 were cultured in Leibowitz’s L-15 medium. NCM460 were cultured in F-12 medium. HCT-8, HCT116, LOVO and RKO cells were cultured in RPMI-1640. All cells were cultured with 10% fetal bovine serum (FBS, Gibco; Thermo Fisher Scientific, Inc., Waltham, MA, United States) and 100 U/ml penicillin (Sigma-Aldrich; Merck KGaA), and 100 μg/ml streptomycin (Sigma-Aldrich; Merck KGaA) at 37°C with 5% CO_2_.

## Cell Transfection and Infection

Cell transfection was achieved through miRNA mimics and siRNAs to interfere the expression level of miR-654-3p and SRC which were purchased from Guangzhou RiboBio Co., Ltd., And final concentration of 50 nM was applied for transient transfection. Transfection procedure was carried out with Lipofectamine 3000 (Invitrogen, Carlsbad, CA, United States) according to the instructions.

For *in vivo* studies, lentivirus vector LV-GFP-Puro (Shanghai GeneChem Co., Ltd., Shanghai, China) overexpressing miR-654-3p (LV-miR-654-3p) or the negative control sequence were applied to infect HCT116 cells and stabilized by puromycin antibiotic selection applied for 7 days with a concentration of 0.6 μg/ml.

### qRT-PCR

The two step qRT-PCR were performed with PrimeScript RT reagent kit and SYBR Green PCR Master Mix (Takara Bio, Inc., Otsu, Japan) according to manufacturer protocol at CFX Real-Time System (Bio-Rad Laboratories, Inc., CA, United States). By applying the 2^–ΔΔ*Ct*^ method, the data of RNA expression level were normalized to housekeeping gene GAPDH or U6 for mRNAs and miRNAs, respectively. The primer sequences were: miR-654-3p forward, 5′- TATGTCTGCTGACCATCACCTT -3′; U6 forward, 5′-CTCGCTTCGGCAGCACA-3′; SRC forward, 5′-TGGCAAGATCACCAGACGG-3′ and reverse, 5′- GGCACC TTTCGTGGTCTCAC-3′; GAPDH forward, 5′- CACCCACTC CTCCACCTTTG -3′ and reverse, 5′-CCACCACCCTGTTGC TGTAG-3′.

### Western Blot

Cell lysis were prepared with RIPA (Solarbio Co., Ltd., Beijing, China). Equal quantities of protein (20 μg/lane) were separated by Tris-glycine poly acrylamide gels and transferred to polyvinylidene fluoride membranes (Sigma-Aldrich; Merck KGaA). Membranes were blocked by 5% non-fat milk resolved in Tris-buffer saline and incubated with primary antibodies at 4°C overnight followed by horseradish peroxidase-labeled secondary antibody. Visualized using enhanced chemiluminescence (Pierce; Thermo Fisher Scientific Inc.). Primary antibodies were: anti-SRC (1:1,000; cat. no. 2109; Cell Signaling Technology, Inc., Danvers, MA, United States) and anti-GAPDH (1: 1,000; cat. no. 5174; Cell Signaling Technology, Inc., Danvers, MA, United States) served as a loading control.

### Cell Proliferation Assay

Cell proliferation assay were performed by cell counting kit-8 (CCK8). Transfected cells (SW480 and HCT116) were seeded into 96-well plates at 1000 cells/well and detected at 24, 48, 72, and 96 h. viability of cells were calculated on a microplate reader (Bio-Rad Laboratories, Inc.) at 450 nm. Cells in each group were tested for 4 replicates and each assay was examined 3 times.

### Colony Formation Assay

For Colony formation assay, cells were seeded into 6-well plates at 500 cells/well at 37°C following transfection. Cells were incubated for 14 days and fixed with 4% paraformaldehyde then stained with 0.1% crystal violet. The count of the colonies was calculated and analyzed by Image J software. Duplicate assays were carried out three times.

### Cell Migration Assay

Cell migration and invasion assays were performed by transwell and wound healing assay. Transwell assay, a total of 5 × 10^5^ cells were seeded into upper chambers with 8 μm pore size membrane and medium supplemented with 30% FBS as a chemoattractant in the lower chambers. The cells were incubated for 48 h and fixed with 4% paraformaldehyde for 15 min and continually stained with 0.1% crystal violet for 15 min at room temperature. Cells in the upper chambers were gently removed by a cotton swab and observed with inverted microscope (magnification, x200; Leica DM IL LED; Leica Microsystems GmbH, Wetzlar, Germany). For wound healing assay was performed in 6-well plates. A total of 1 × 10^6^ cells were seeded into 6-well plates. The scratch were made by the 100 μl pipette tip when the cell aggregation reached 85-90%. The scratches were recorded at once and at 24, 48, and 72 h in the same spot. The images were analyzed by Image J software. Duplicate assays were carried out three times.

### Cell Invasion Assay

Cell migration and invasion assays were performed by transwell assay. A total of 5 × 10^5^ cells were seeded into upper chambers with Matrigel pre-coated 8 μm pore size membrane and medium supplemented with 30% FBS as a chemoattractant in the lower chambers. The cells were incubated for 72 h00 and fixed with 4% paraformaldehyde for 15 min and continually stained with 0.1% crystal violet for 15 min at room temperature. Cells in the upper chambers were gently removed by a cotton swab and observed with inverted microscope. Duplicate assays were carried out three times.

### Flow Cytometry Assay

The CRC cells were stained with FITC Annexin V Apoptosis Detection Kit (BD Biosciences, Franklin Lakes, NJ, United States) and with Cycletest^TM^ plus DNA kit (BD Biosciences, Franklin Lakes, NJ, United States) to gain the apoptosis analysis and cell cycle analysis, respectively, according to the manufacturer’s instructions. Data was analyzed with FlowJo (version 7.0; Tree Star, Inc., Ashland, OR, United States) on a flow cytometer (BD Biosciences).

### Luciferase Reporter Assay

The binding site between miR-654-3p and mRNA of SRC was predicted by Target Scan Human^1^ and prepared by Sangon Biotech. Plasmid of WT and MUT for SRC (500 ng/well) and miR-654-3p NC or mimic (100 nM/well) were co-transfected in 24-well plates (1 × 10^5^ cells/well). After 48 h culturing, firefly to renilla luciferase activities were detected using the Dual-Luciferase reporter assay system (Promega Corporation, Madison, WI, United States). Duplicate assays were carried out three times.

### Xenograft Mice Model

For tumorigenesis assays, 10 female, 4–6 weeks of age BALB/c-nude mice (Vital River Laboratories, Beijing, China) were randomly assigned to two groups. 5 × 10^6^ cells were injected to right flank of each mouse subcutaneously. The volume of the tumors was calculated every four days in accordance with the formula: V = (length × width^2^)/2. The mice were scarified 40 days after injection continue with tumor extraction for volume measurements. The mice were raised in accordance with guidelines provided by the Institutional and Animal Care and Use Committee. Permission from the Animal Research Committee of the Peking University People’s Hospital (Beijing, China) was received for all animal experiments.

### Statistical Analysis

Data were represented the mean ± SD. and analyzed with SPSS 23.0 software (SPSS Inc., Chicago, IL, United States). *P* < 0.05 was considered to be statistically significant. The associations of miR-654-3p expression levels with clinicopathologic parameters of CRC patients were measured by Pearson χ2 test. Spearman’s correlation was employed to assess the association between the expression of miR-654-3p and SRC. The difference of miR-654-3p or SRC levels between para cancerous and normal CRC tissues were evaluated using paired *t* test. Cox regression analysis was employed for single factor and multivariate analysis for variables affecting overall survival. Overall survival of two groups patient determined by the expression level of miR-654-3p was analyzed by the Kaplan-Meier method.

## Results

### Downregulation of miR-654-3p in Specimens Indicates Poor Prognosis of CRC Patients

**FIGURE 1:**
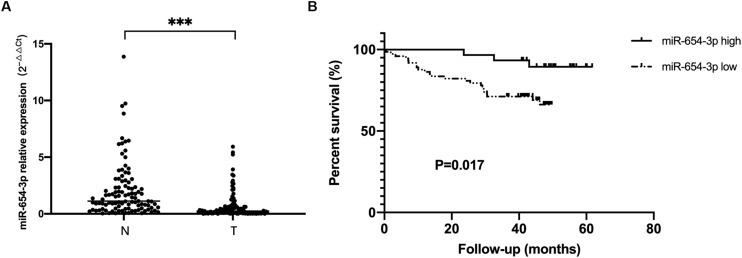
miR-654-3p downregulation in colorectal cancer indicates poor prognosis. **(A)** Relative expression of miR-654-3p of matched colorectal cancer samples and adjacent normal specimens (*n* = 103), assessed by reverse transcription-quantitative polymerase chain reaction. **(B)** Kaplan-Meier survival curves of CRC cases with overexpressed (*n* = 30) and reduced (*n* = 73) miR-654-3p. ***p < 0.005 assessed using log-rank test. N, adjacent normal colorectal samples; T, CRC tissue specimens.

The expression levels of miR-654-3p were measured by qRT-PCR in 103 pairs of matched CRC tissues and adjacent normal tissues, independently. The results exhibited that miR-654-3p was remarkably reduced in CRC tissues as compared to adjacent normal tissues (*P* < 0.05, Figure 1A). Suppressed miR-654-3p levels were highly associated with distant metastasis. However, age, sex, tumor size, tumor differentiation, lymph node metastasis, AJCC stage, and microsatellite stability did not show statistical differences between the two groups (Table 1). Furthermore, cases expressing a low level of miR-654-3p presented poorer prognosis as opposed to those who showed high expression of the miRNA (*P* < 0.05, Figure 1B). Single-factor analysis demonstrated that tumor differentiation, lymph node metastasis, distant metastasis, AJCC stage, and miR-654-3p were closely associated with the overall survival (Table 2). In multivariate analysis, only distant metastasis exhibited statistical significance (Table 2).

**TABLE 1:** Association between miR-6654-3p and clinicopathologic characters in CRC.

	miR-654-3p expression			
Parameters	High (*n* = 30)	Low (*n* = 73)	Total (*n* = 103)	*P*-value
**Age**				
≤60	12	25	37	0.580
>60	18	48	66	
**Sex**				
Male	16	42	58	0.696
Female	14	31	45	
**Tumor size, cm**				
≤4	17	31	48	0.189
>4	13	42	55	
**Tumor differentiation**				
Well/moderate	26	59	85	0.345
Poor	4	14	18	
**Lymph node metastasis**				
Negative	21	41	63	0.192
Positive	9	32	41	
**Distance metastasis**				
Negative	30	64	94	0.044*
Positive	0	9	9	
**AJCC stage**				
I + II	21	39	60	0.192
III + IV	9	34	43	
**Microsatellite stability**				
MSI	5	15	20	0.439
MSS	25	58	83	

*P < 0.05. miRNA, microRNA; MSI, Microsatellite instability; MSS, Microsatellite stability.

**TABLE 2:** Single factor analysis and multivariate analysis of parameters associated with overall survival in patients with CRC.

	Single-factor analysis		Multivariate analysis	
Variables	HR (95% CI)	*P*-Value	HR (95% CI)	*P*-Value
Age	0.838 (0.380–1.847)	0.661		
Sex	1.058 (0.472–2.375)	0.891		
Tumor size	1.866 (0.831–4.187)	0.131		
Tumor differentiation	2.724 (1.181–6.282)	0.019*	2.022 (0.840–4.864)	0.116
Lymph node metastasis	4.239 (1.841–9.749)	0.001*	1.913 (0.218–16.805)	0.559
Distant metastasis	6.689 (2.786-16.060)	0.000*	2.823 (1.006–7.926)	0.049*
AJCC stage	4.771 (2.003–11.365)	0.000*	0.336 (0.097–1.162)	0.085
Microsatellite stability	0.688 (0.377–1.255)	0.233		
miR-654-3p	0.255 (0.076–0.852)	0.026*	1.773 (0.164–18.269)	0.647
SRC	1.978 (0.858–4.559)	0.109		

*p < 0.05. CI, confidence interval; HR, hazard ratio; miRNA, microRNA.

### miR-654-3p Inhibits CRC Cells Proliferation, Migration and Invasion

**FIGURE 2:**
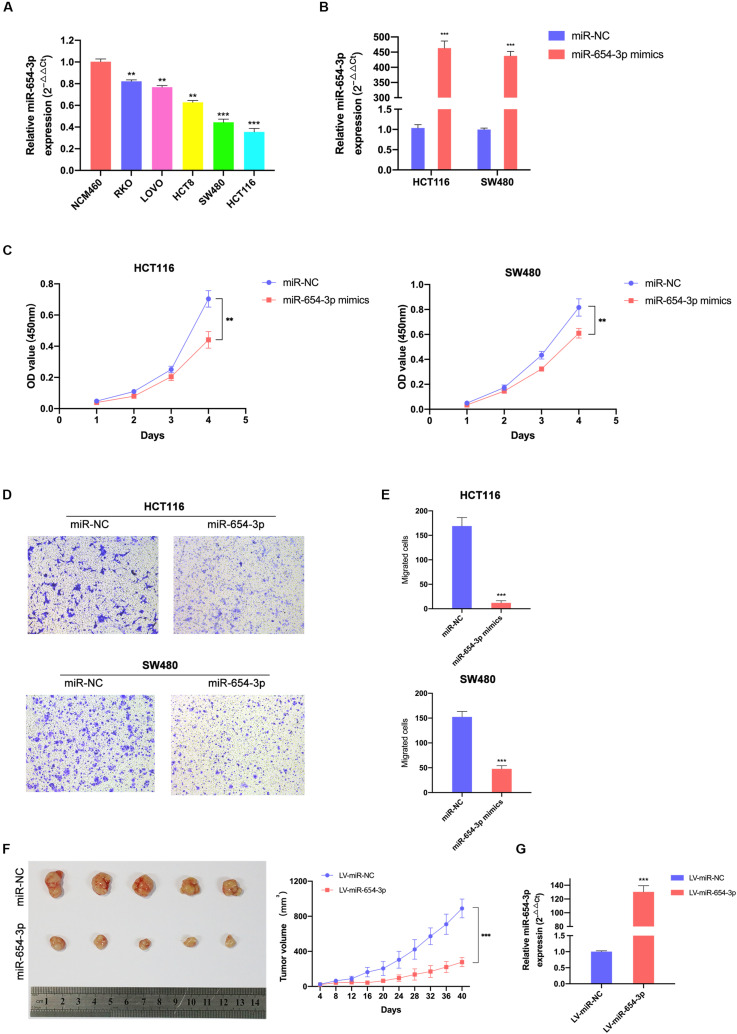
Elevated miR-654-3p inhibits CRC cells proliferation and migration *in vitro* and in mice. **(A)** Relative expression levels of miR-654-3p in 6 colorectal cell lines analyzed by qRT-PCR. **(B)** Relative expression levels of miR-654-3p in HCT116 and SW480 transfected with miR-NC or miR-654-3p. **(C)** Growth curves of HCT116 and SW480 following the overexpression of miR-654-3p as assessed by Cell Counting Kit-8 assay. **(D)** Transwell assay HCT116 and SW480 following the overexpression of miR-654-3p (magnification, x200). **(E)** Statistical analysis of the transwell assay results. **(F)** Effects of miR-654-3p on the growth of xenograft CRC tumor in mouse models. Tumor growth curves were generated by assessing tumor volumes at 4-day intervals. **(G)** Relative expression levels of miR-654-3p in xenograft CRC tumor in mouse models measured after the tumor extraction and volume measurements. The error bars represent the means ± SDs from three independent experiments. **p < 0.01, ***p < 0.005.

To further explore whether miR-654-3p affects the biological behavior of CRC cells, proliferation, migration and invasion assays were performed. First, we detected the expression levels of miR-654-3p in various CRC cell lines using qRT-PCR analysis and found that HCT116 and SW480 exhibited low expression of miR-654-3p (Figure 2A). Next, miR-654-3p mimics were transfected to upregulate the low expression of miR-654-3p in HCT116 and SW480 cells (Figure 2B). As shown in Figure 2C, compared to NC controls, the cell proliferation capacity of HCT116 and SW480 cells was remarkably suppressed. Transwell assays demonstrated that the migrate ability of miR-654-3p mimic groups was suppressed in HCT116 and SW480 cells (Figures 2D,E). And the invasive capacity of miR-654-3p mimic groups was declined in HCT116 and SW480 cells (Supplementary Figures S1A,B).

To further confirm the results of *in vitro* tests, the upregulation of miR-654-3p subcutaneous xenograft growth *in vivo* was studied. The volume of tumors of the miR-654-3p overexpressed group increased slower than that of the NC group (Figure 2F). As expected, the expression level of miR-654-3p was higher in the miR-654-3p-overexpressed group, as assessed by qRT-PCR (Figure 2G).

**FIGURE 3:**
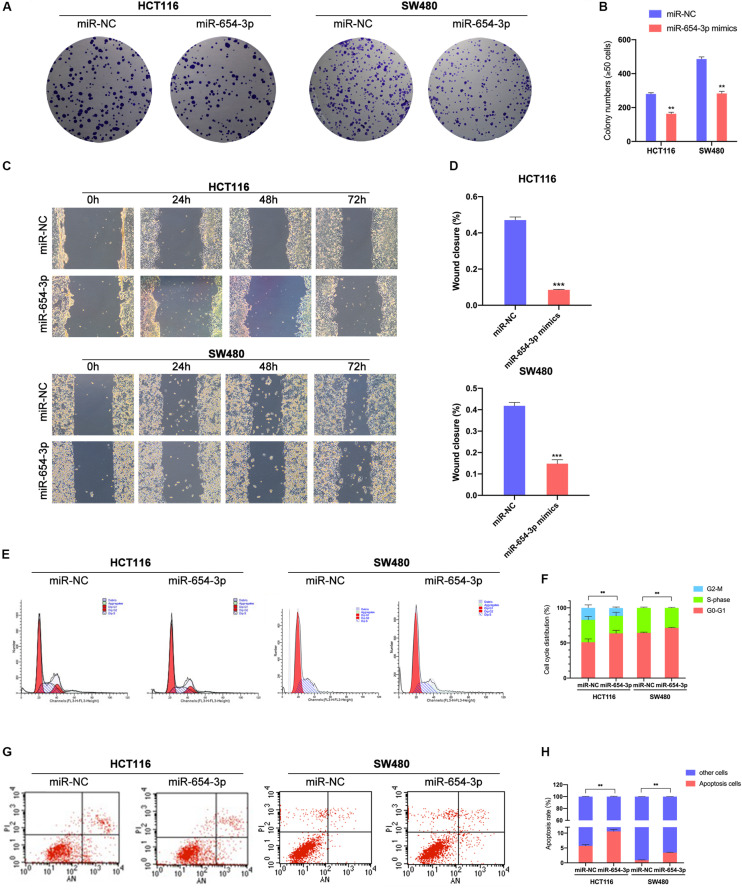
Upregulation of miR-654-3p suppressed migration and promotes G0/G1 phase arrest and apoptosis in CRC. **(A)** Representative images of the colony formation assay following the overexpression of miR-654-3p in HCT116 and SW480. **(B)** Statistical analysis of the colony formation assay results. **(C)** Wound healing assay showing the differences in migration capacities in the indicated cells at 4 regular intervals. **(D)** Statistical analysis of the wound healing assay results. **(E)** Cell cycle distribution of HCT116 and SW480 was assessed by flow cytometry following treatment with miR-NC or miR-654-3p. **(F)** Upregulated miR-654-3p promoted cell cycle arrest at the G0/G1 phase. **(G)** Apoptosis rates were detected by flow cytometry with transfection of miR-654-3p or miR-NC. **(H)** Overexpressed miR-654-3p led to higher rate of apoptosis in HCT116. The error bars represent the means ± SDs from three independent experiments. **p < 0.01, ***p < 0.005.

Colony formation assay demonstrated that miR-654-3p suppressed the proliferation of HCT116 and SW480 cells (Figures 3A,B) as compared to the NC groups. Wound healing assay revealed that the overexpression of miR-654-3p suppressed the proliferative and migration capacities of HCT116 and SW480 cells (Figures 3C,D). Furthermore, the upregulated miR-654-3p cells were blocked in G0/G1 phase as compared to the miR-NC controls in HCT116 and SW480 (Figures 3E,F). It also resulted in a high apoptosis rate in HCT116 and SW480 cells (Figures 3G,H).

### miR-654-3p Acts by Directly Targeting SRC

**FIGURE 4:**
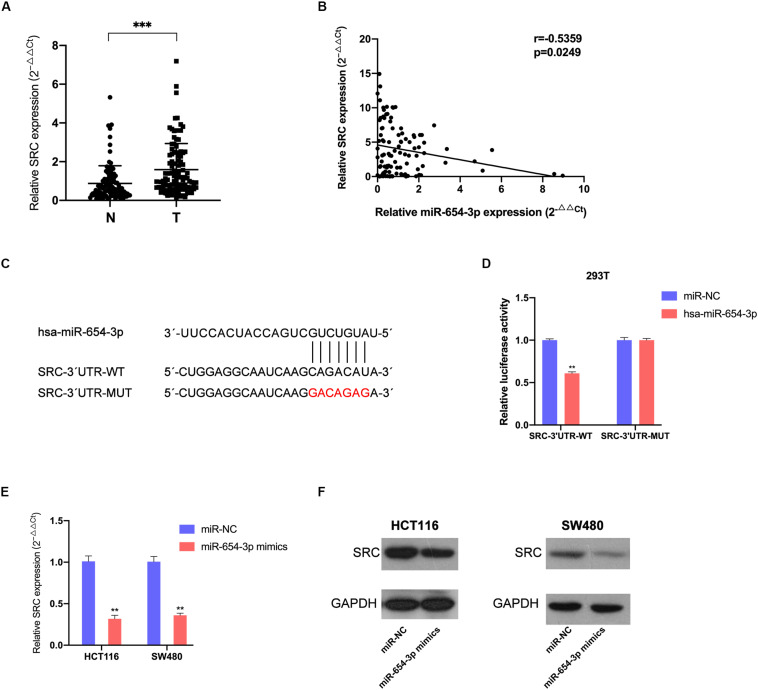
MiR-654-3p acts by directly targeting SRC. **(A)** Elevated expression levels of SRC were detected in CRC samples compared with adjacent normal specimens (*n* = 103). **(B)** The association of SRC relative expression levels with miR-654-3p using Pearson’s correlation analysis. **(C)** The predicted binding site and the mutated binding site of the miR-654-3p target on the 3′-UTR of SRC. **(D)** Luciferase activity assay showing that miR-654-3p influenced the luciferase activity of SRC 3′-UTRs in 293T. **(E)** Effects of miR-654-3p upregulation on SRC expression at mRNA level. **(F)** Effects of miR-654-3p upregulation on SRC expression at protein level. The error bars represent the means ± SDs from three independent experiments. *P < 0.05, **p < 0.01, ***p < 0.005. NC, negative control; UTR, untranslated region; WT, wild-type; MUT, mutated.

To locate the downstream target protein of miR-654-3p, which supports its function, we used TargetScan 7.2 to predict the potential targets; the database predicted SRC was the target of miR-654-3p. The association of miR-654-3p with SRC expression was detected in CRC and matched adjacent normal colorectal tissue specimens. The SRC expression levels were remarkably elevated in CRC cells as compared to normal colorectal tissues (Figure 4A) and elevated SRC levels were highly associated with distant metastasis (Supplementary Table S1). Moreover, the expression level of SRC was negatively associated with miR-654-3p using Pearson’s correlation analysis (Figure 4B).

Dual-luciferase reporters contained the 3′-UTR fragments of SRC with the miR-654-3p binding sites or mutant fragments (Figure 4C) that were co-transfected with miR-654-3p mimics or NC mimics. The relative luciferase activity of the mutant group did not exhibit any significant changes, while that was remarkably reduced in the wild-type group (Figure 4D). The upregulation of miR-654-3p established a negative correlation between miR-654-3p and SRC at both mRNA and protein levels in HCT116 and SW480 cells (Figures 4E,F).

### Downregulated SRC Suppressed CRC Cells Proliferation, Migration and Invasion

**FIGURE 5:**
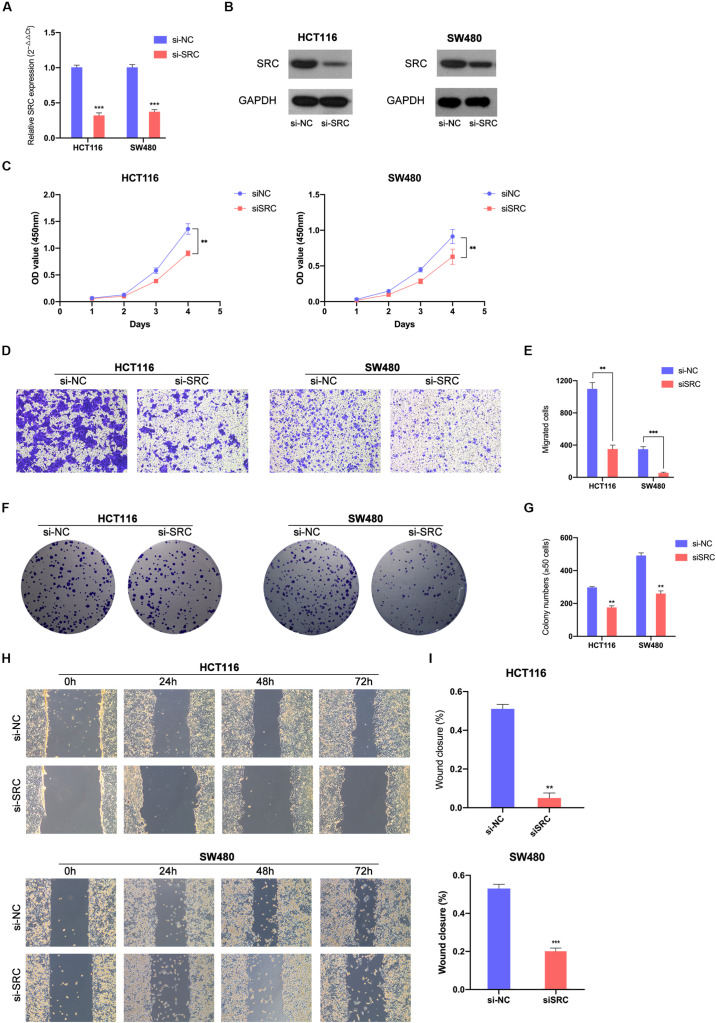
Downregulated SRC suppressed CRC cells proliferation and migration. Relative expression levels of SRC in HCT116 and SW480 transfected with si-NC or siSRC at mRNA level **(A)** and protein level **(B)**. **(C)** Downregulated SRC suppressed cell growth in HCT116 and SW480 by CCK-8 assay. **(D, E)** Transwell assay showed decreased expression of SRC declined the migrate abilities in HCT116 and SW480 (left, magnification, x200); statistical analysis of the transwell assay results (right). **(F, G)** Representative images of the colony formation assay following the downregulation of SRC (left); statistical analysis of the colony formation assay results (right). **(H, I)** Decreased expression level of SRC weaken the migration capacities detected by Wound healing assay (left); Statistical analysis of the wound healing assay results (right). The error bars represent the means ± SDs from three independent experiments. *P < 0.05, **p < 0.01, ***p < 0.005.

To further demonstrate the effect of the miR-654-3p/SRC pathway, function-based experiments were performed in SRC-downregulated CRC cells (Figures 5A,B). The CCK8 analysis showed that the cell proliferation ability was weak in SRC-downregulated groups in HCT116 and SW480 cells (Figure 5C). In the colony formation assay, miR-654-3p mimic groups in HCT116 and SW480 cells formed fewer colonies as compared to the NC groups (Figures 5F,G). Moreover, suppressed migration (Figures 5D,E) and invasion (Supplementary Figures S1C,D) capacity was detected in Transwell assay and wound healing assay (Figures 5H,I) of miR-654-3p mimic groups in HCT116 and SW480 cells.

### Upregulation of SRC Neutralized the Suppression of miR-654-3p

**FIGURE 6:**
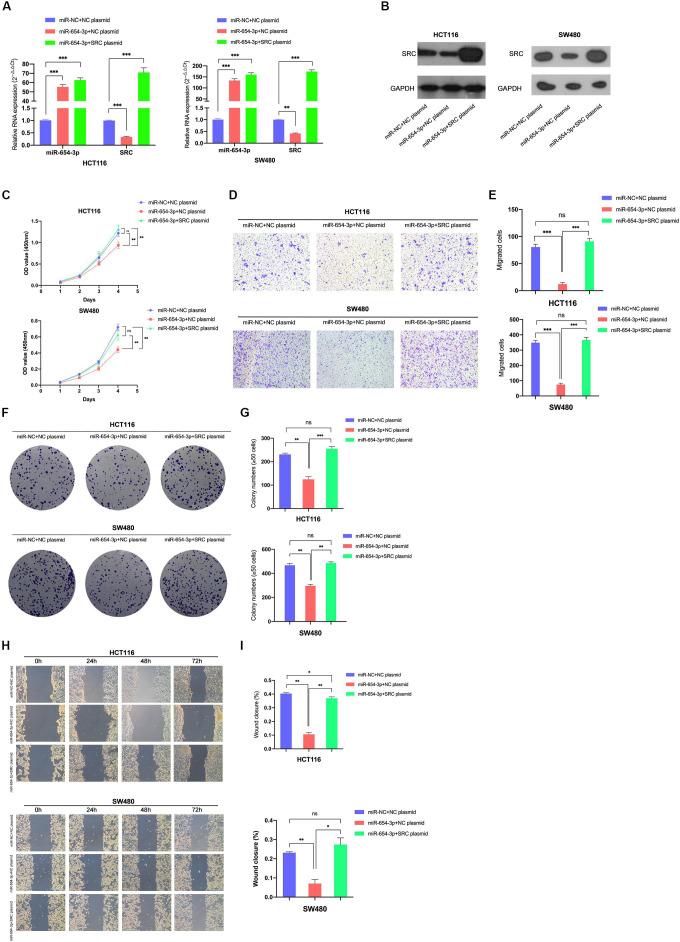
Reversal effect on proliferation and migration by upregulating miR-654-3p and SRC simultaneously. Relative expression levels of miR-654-3p and SRC in HCT116 and SW480 transfected with miR-NC + NC plasmid, miR-654-3p + NC plasmid or miR-654-3p + SRC plasmid at mRNA level **(A)** and protein level **(B)**. **(C)** Growth curves of HCT116 and SW480 following the transfection assessed by Cell Counting Kit-8 assay. **(D, E)** Transwell assay in HCT116 and SW480 following the transfection (left, magnification, x200); Statistical analysis of the transwell assay results (right). **(F, G)** Representative images of the colony formation assay following the transfection in HCT116 and SW480 (left); Statistical analysis of the colony formation assay results (right). **(H, I)** Migration capacities detected by Wound healing assay (left); Statistical analysis of the wound healing assay results (right). The error bars represent the means ± SDs from three independent experiments. *P < 0.05, **p < 0.01, ***p < 0.005.

For further verification of the role of SRC in the miR-654-3p-associated anti-cancer mechanism, a rescue experiment of SRC was evaluated by upregulating miR-654-3p and SRC at the same time in HCT116 and SW480, respectively (Figures 6A,B). The suppression effect on the proliferation capacity effectuated by miR-654-3p upregulation was distinctly reversed after the SRC level was elevated (Figure 6C). HCT116-miR-654-3p + SRC and SW480-miR-654-3p + SRC exhibited enhanced proliferation, migration and invasion capacities in Transwell assay (Figures 6D,E and Supplementary Figures S1E,F), colony formation assay (Figures 6F,G) and wound healing assay (Figures 6H,I), which was consistent with the results of the CCK8 assay.

## Discussion

miRNAs are crucial regulators of gene expression and promising candidates for biomarker development in various malignancies (Iorio and Croce, 2012; Dong et al., 2013). miR-654-3p has been reported as a tumor suppressor in gastric cancer (Deng et al., 2020), prostate cancer (Formosa et al., 2014), and hepatocellular carcinoma (Yang et al., 2020). However, the roles of miR-654-3p in tumorigenesis, cell proliferation, and migration are unclear.

Deng et al. (2020) reported that miR-654-3p acts as a promoter in gastric cancer, while Zhou et al. (2020) demonstrated that miR-654-3p suppresses the proliferation ability by targeting SYTL2 in osteosarcoma. In the present study, functional experiments indicated that the upregulation of miR-654-3p in CRC cell lines suppressed cell proliferation and migration capacities. The overexpressed miR-654-3p induced G0/G1 phase arrest, high rate of apoptosis, and less tumorigenesis in nude mice. The *in vivo* experiments confirmed that the overexpression of miR-654-3p suppressed the growth of CRC xenograft tumors in nude mice. These findings strongly indicated that miR-654-3p inhibits tumor proliferation and invasion in CRC.

miRNAs affect the malignant cell proliferation, migration and invasion capacities at post-transcriptional regulation level via binding to the 3′-UTR of the target mRNA (Yu et al., 2018; Ali Syeda et al., 2020). Reportedly, miR-654-3p targets several genes associated with malignancy, such as P21 in gastric cancer (Deng et al., 2020), AKT3 in ovarian cancer (Duan et al., 2020), and SYTL2 in osteosarcoma (Zhou et al., 2020). SRC is predicted to be directly targeted by miR-654-3P.

High expression of miR-654-3p decreased the SRC levels in CRC specimens while low levels were detected with high levels of SRC in paired non-cancerous tissues detected by qRT-PCR. A similar conclusion was deduced by upregulating miR-654-3p in CRC cell lines. Bioinformatics and the dual-luciferase reporter assays confirmed the direct binding of miR-654-3p to the 3′-UTR of SRC mRNA. The rescue experiment verified the conclusion. The suppression of cell proliferation migration and invasion of the cells by upregulated miR-654-3p was remarkably reversed by overexpression of SRC simultaneously.

Metastasis, the major cause of death in CRC, results from multi-step processes, including angiogenesis, invasion, circulation, extravasation, and metastatic colonization (Tauriello et al., 2017; Qi and Ding, 2018). Accumulating evidence demonstrated that miRNA plays a crucial role in metastasis (Guo et al., 2017; Hur et al., 2017; Balacescu et al., 2018; Ozawa et al., 2018). Therefore, the mechanism of miRNA underlying CRC metastasis might generate a miRNA-based target for diagnosis and therapeutics of CRC (Ji et al., 2018; Li et al., 2018; Zhao et al., 2020). As mentioned above, the expression level of miR-654-3p is closely related to distant metastasis. Moreover, the overexpression of miR-654-3p resulted in significant suppression of the migration of CRC cells. These results indicated that miR-654-3p might be a novel target for CRC therapeutics, especially in metastatic cases.

SRC one of the SRC family kinases (SFKs) is involved in aggressive tumor proliferation, migration, and invasion in numerous malignancies (Yeatman, 2004; Martellucci et al., 2020). Meanwhile, SRC was elevated and activated in CRC cell lines and CRC tissue, thereby contributing to its malignant behavior (Tan et al., 2019; Jin, 2020). The SRC downstream signal pathways, such as protein tyrosine phosphatase α (PTPα), SH-containing phosphatases SHP1/SHP2, and nuclear factor-kappa B (NF-κB) pathway indicate a vital role of SRC in occurrence and development of CRC (Aleshin and Finn, 2010; Brady et al., 2011). The mechanism of miR-654-3p affects malignant behavior of CRC cells is by regulating the expression levels of SRC.

In summary, the present study demonstrated that human CRC tissues exhibit decreased miR-654-3p expression as compared to para-cancerous normal tissues, which is associated with poor prognosis of CRC patients. miR-654-3p suppressed cell proliferation, migration and invasion capacities in CRC cell lines and nude mice by directly targeting SRC. Thus, these findings provide a novel miR-based molecular target for CRC therapy.

## Biosecurity Statement

All standard biosecurity and institutional safety procedures have been adhered to in all the experiment procedures in this article.

## Data Availability Statement

The analyzed datasets generated during the study are available from the corresponding author on reasonable request.

## Ethics Statement

The studies involving human participants were reviewed and approved by Research Ethics Committee of Peking University. The patients/participants provided their written informed consent to participate in this study. The animal study was reviewed and approved by Animal Research Committee of the Peking University People’s Hospital.

## Author Contributions

HZ, YY, and BW were responsible for the study design, drafting and editing of the original article, data acquisition and data analysis. HZ, MZ, and YZ performed the experiments. HZ, QW, and ZZ were responsible for data acquisition and analysis. ZS, KJ, and SW were responsible for data interpretation and methodology. YY, BW, and SW were responsible for supervision. HZ, BW, ZS, and YY revised the manuscript. All authors have read and approved the final manuscript.

## Conflict of Interest

The authors declare that the research was conducted in the absence of any commercial or financial relationships that could be construed as a potential conflict of interest.

## Footnotes

1 http://www.targetscan.org/vert_72/

## Supplementary Material

FIGURE S1 miR-654-3p affection on the invasion capacity of Crc cells. **(A, B)** Transwell assay Hct116 and Sw480 following the overexpression of miR-654-3p (left, magnification, x200); statistical analysis of the transwell assay results (right). **(C, D)** Transwell assay showed decreased expression of Src declined the invasive abilities in Hct116 and Sw480 (left, magnification, x200); statistical analysis of the transwell assay results (right). **(E, F)** Transwell assay in Hct116 and Sw480 following the transfection (left, magnification, x200); Statistical analysis of the transwell assay results (right).
