# Supplementary figures and images for: Downregulation of miR-654-3p in Colorectal Cancer Indicates Poor Prognosis and Promotes Cell Proliferation and Invasion by Targeting SRC
Source: Front Genet. 2020 Sep 30;11:577948. doi: 10.3389/fgene.2020.577948 (PMC7554538; doi:10.3389/fgene.2020.577948)

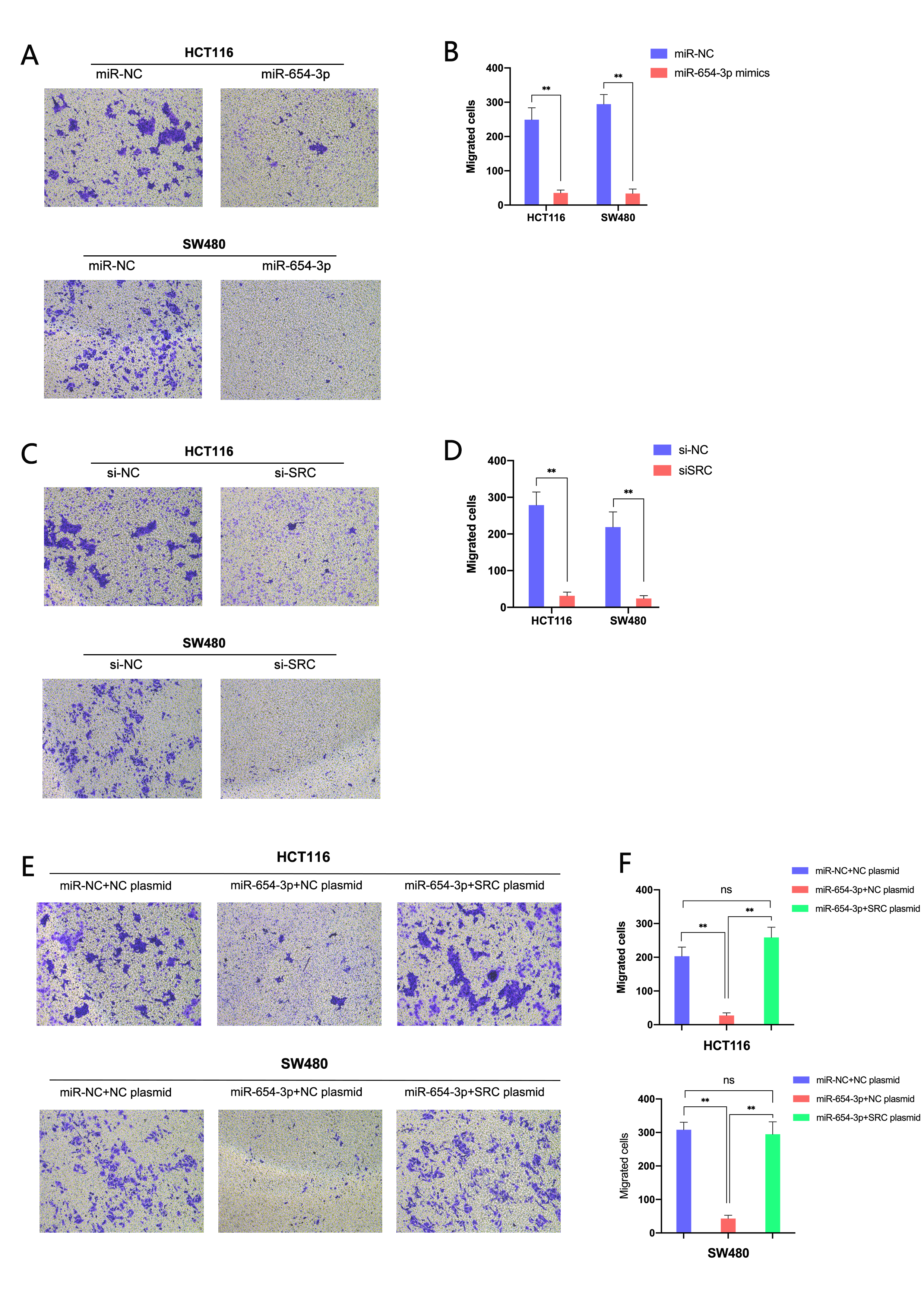

Supplement: FIGURE S1 — miR-654-3p affection on the invasion capacity of Crc cells. (A, B) Transwell assay Hct116 and Sw480 following the overexpression of miR-654-3p (left, magnification, x200); statistical analysis of the transwell assay results (right). (C, D) Transwell assay showed decreased expression of Src declined the invasive abilities in Hct116 and Sw480 (left, magnification, x200); statistical analysis of the transwell assay results (right). (E, F) Transwell assay in Hct116 and Sw480 following the transfection (left, magnification, x200); Statistical analysis of the transwell assay results (right). [file Image_1.TIF]
