# Supplementary material for: Downregulation of miR-654-3p in Colorectal Cancer Indicates Poor Prognosis and Promotes Cell Proliferation and Invasion by Targeting SRC
Source: Front Genet. 2020 Sep 30;11:577948. doi: 10.3389/fgene.2020.577948 (PMC7554538; doi:10.3389/fgene.2020.577948)
Supplement: Supplementary file 2 [file Table_1.docx]

| Table S1. Association between SRC and clinicopathologic characters in CRC | | | | |
| --- | --- | --- | --- | --- |
|  | SRC expression | | | |
| Parameters | High(n=59) | Low(n=44) | Total(n=103) | P-value |
| Age |  |  |  |  |
| ≤60 | 22 | 15 | 37 | 0.738 |
| ＞60 | 37 | 29 | 66 |  |
| Sex |  |  |  |  |
| Male | 36 | 22 | 58 | 0.265 |
| Female | 23 | 22 | 45 |  |
| Tumor size,cm |  |  |  |  |
| ≤4 | 26 | 22 | 48 | 0.550 |
| ＞4 | 33 | 22 | 55 |  |
| Tumor differentiation |  |  |  |  |
| Well/moderate | 46 | 39 | 85 | 0.158 |
| Poor | 13 | 5 | 18 |  |
| Lymph node metastasis |  |  |  |  |
| Negative | 33 | 29 | 63 | 0.306 |
| Positive | 26 | 15 | 41 |  |
| Distance metastasis |  |  |  |  |
| Negative | 51 | 43 | 94 | 0.049^*^ |
| Positive | 8 | 1 | 9 |  |
| AJCC stage |  |  |  |  |
| I+II | 31 | 29 | 60 | 0.174 |
| III+IV | 28 | 15 | 43 |  |
| Microsatellite stability |  |  |  |  |
| MSI | 14 | 6 | 20 | 0.200 |
| MSS | 45 | 38 | 83 |  |
| ^*^P<0.05. miRNA, microRNA; MSI, Microsatellite instability; MSS, Microsatellite stability | | | | |
